# “I Am Not Just a Prisoner”: the effects of social identification on well-being in Italy

**DOI:** 10.3389/fpsyg.2024.1466376

**Published:** 2024-12-12

**Authors:** Cristina O. Mosso, Alessandra Caldera, Camilla Re

**Affiliations:** Department of Psychology, University of Turin, Torino, Italy

**Keywords:** stigma, social identification, well-being, prisoner, self-regulation

## Abstract

Recent research on social cures has posited that one’s social identity associated with group membership can promote adjustment and affect well-being, especially for vulnerable individuals. In this study, we argue that, although the negative consequences of group-based stigma affect prisoners’ well-being, having multiple social identities can protect them and mitigate the detrimental effects of stigma. The results of this cross-sectional study reveal that group identification with prisoners (*N* = 52) was negatively associated with well-being and anticipated discrimination. In contrast, education was positively associated with self-regulation and life satisfaction. Group identification with prisoners threatens self-regulation when the salience is controlled. We discuss the significance of our findings in the context of stigma and resocialization.

## 1 Introduction

Those who have experienced prison life generally described it as a change in the self, marked primarily by the deprivation of freedom and privacy. Thus, living in prison means being placed in a world of one’s own, with its own rules, conduct, and dynamics that, with the goals of re-education and re-socialization, accompany inmates as they construct new social identities. Furthermore, according to the psychosocial social care approach (Haslam et al., 2018), adaptation to such changes has implications for inmates’ relationships and health (Jetten et al., 2017; Kyprianides et al., 2019). The social groups belonging can have beneficial consequences for well-being and adjustment even within the most vulnerable populations especially for those who are stigmatized (Gleibs et al., 2011; Cruwys et al., 2014; Johnstone et al., 2015). The purpose of this study is to provide empirical evidence on the relationship between identification with a stigmatized group and well-being among inmates in an Italian prison center, where education has been promoted as a form of rehabilitation.

The Italian prison system is facing critical issues compared to those in other European countries (WHO, 2003), including overcrowding. According to recent data (updated as of March 3, 2024), the Italian territory has 61,049 inmates across 190 penitentiary institutions, with the overall system operating at 117.2% of its design capacity. Furthermore, Italian prisons are severely understaffed, and consequently, so are programs meant to provide opportunities for social recovery.

Since Law No. 395/1990 was introduced, prison police in Italy have been required to maintain order first and then implement rehabilitation programs (Baudino, 2014). Moreover, they are responsible for inmate rehabilitation (Santorso, 2021). The problems in the Italian prison system, combined with political pressure from prison staff unions and human rights organizations, have long indicated the need for educational programs that facilitate social reintegration and can provide support to these individuals in decision-making and job opportunities, which those with criminal records often find unattainable. Although in the literature, especially the studies on desistance and disengagement focused on the process of change and the related psychosocial processes (McNeil, 2016) at a mainly individual level, often disconnected from the social groups implications in which the desistance take place (Weaver, 2019). In a psychosocial perspective, several studies have shown that the positive correlation between self-efficacy and education can catalyze identity change, fostering positive identity transitions by encouraging agency (Lerman and Sadin, 2023). As a protective factor of identity, access to education can greatly affect prisoners’ self-concept and promote well-being and self-efficacy. Specifically, for those experiencing imprisonment, education can help them transition from a negative to a positive self-concept (social identity change).

### 1.1 Social identity and well-being

Lewin (1948) argued that life changes may affect individuals’ sense of security and fear of being unable to earn a living, which in turn will affect their grounding, continuity, and stability. According to Lewin, such grounding is provided by social groups; thus, changes force individuals to reorient the personal self as well as their connections to others. These assumptions have been explored, reworked, and incorporated into the theory known as the social identity model of identity change (SIMIC), according to which people can define their sense of self either in social or personal terms (“us” and “we,” not only “I” and “me”). It shows how a group can act not only as a support and social cure but also as a social curse, especially when confronted with the outside world. Based on the theory of social identity (Tajfel and Turner, 1979, 1986), a key assumption of this theory is that group membership, defined as social identity, contributes to one’s self-concept and the salience of social identity, thus encouraging individuals to differentiate their ingroup from other groups. The social identity model of identity change (SIMIC) focuses on the effects of life transition on how we define ourselves in relation to others, which helps identify processes that may hinder adjustment following change. Individuals may be particularly reluctant to relinquish a positive identity, especially when that identity is strong (Haslam et al., 2008). Moreover, adjustment depends on an individual’s willingness to become grounded in a new sense of belonging. Taking on a new identity forms the basis for social support and new sources, previous empirical studies showed the beneficial effect of groups on adjustment and well-being during life changes such as the transition to university (Iyer et al., 2009; Cruwys et al., 2021). A longitudinal study of students’ transitions to university (Cruwys et al., 2021; Iyer et al., 2009) further obtained evidence for the role of compatibility between old and new identities, reporting that while students felt some excitement associated with going to university, those who lived away from home for more than 2 months reported symptoms of depression.

Furthermore, recent research on ex-prisoners has analyzed the Kyprianides and Easterbrook (2020) relevance of social identification on well-being and personal satisfaction, showing how identification with prisoners mediates the close relationship between perceived stigma and self-evaluation. Thus, this study aims to adapt the research design of Kyprianides et al. (2019) to collect evidence on the relationship between identification with a stigmatized group and well-being among inmates in Italy in a prison setting where education has been promoted as a resocializing activity for several years. The opportunity to study provides inmates with the chance to build new positive identities, move away from typically negative self-conceptions, and experience higher levels of self-efficacy and well-being. This is possible through “identity displacement.” Another aspect that must inevitably be considered is that social identity is constantly changing, especially at particularly delicate points in individuals’ lives when they can see an upcoming transition. This is the case when, for example, individuals are released from prison and must return to society. The SIMIC highlights this continuous change in social identity and how it is enriched with all the attributes that characterize those who have committed crimes, been punished, and sentenced to a period of imprisonment. Given these theoretical premises, and based on the work of Cruwys et al. (2015), we chose the model of social care for our study.

Research on the effects of stigma has gained renewed attention in recent years (Kyprianides et al., 2019), and a person who has been incarcerated can be aware of and even expect stigmatizing attitudes and acts of discrimination from the public (Feingold, 2021). Sensitivity to and anticipation of such treatment can lead to social withdrawal and a decrease in help-seeking behaviors. However, the rejection-identification model (Branscombe et al., 1999) proposes that rejection and social exclusion by an external group can push members of the stigmatized group to identify more with that group by amplifying cohesion, thereby limiting the negative effect of discrimination. Kyprianides et al. (2019) adopted this model for a study conducted with a sample of 199 former US inmates to assess whether the experience of group-based rejection was indeed associated with greater identification with the “ex-prisoner” group. However, the authors hypothesized that, if that were true, increased identification would lead to a lower level of well-being and therefore not coincide with a better perception of one’s own quality of life, as the rejection-identification model predicts. Therefore, they estimated that identification as a prisoner amplified, rather than reduced, the relationship between rejection and reduced well-being. These results show that both negative and positive changes pose a threat to well-being.

However, decisive factors play a protective role with respect to the degree of well-being of those who leave prison, such as the social network on which they can rely. Thus, assuming that group-based rejection has negative emotional, behavioral, and cognitive consequences that hinder successful reintegration into the community after release, an online survey was conducted. The results show that identification as a former prisoner is key to the identified relationship between group-based rejection and poor well-being.

### 1.2 Aim and hypotheses

Research indicates that group membership can enhance well-being because it satisfies psychological needs (Greenaway et al., 2016). According to the social cure approach, belonging to multiple groups allows individuals to develop more social identities, thereby promoting adaptation, coping, and well-being (Kyprianides et al., 2019). However, in-group identification can also be detrimental to well-being (Wakefield et al., 2019). Prior research has demonstrated that membership in lower-status and disadvantaged minority groups increases the perception of discrimination, thus increasing symptoms of depression, stress, and anxiety in turn (Schmitt et al., 2014), as they are predictors of well-being. Therefore, some people become bearers of concealable stigmatized identities they attempt to hide from others, seeing these identities as socially devalued and linked to negative stereotypes that affect their self-perception of self and well-being (Quinn and Earnshaw, 2013). Based on these assumptions, we propose that prisoners recognize themselves as a heavily stigmatized group and attribute a strong stigma to themselves as well. We posit that those who received an education during their imprisonment will show a greater capacity for self-regulation, identify less with the stigmatized group, and consequently show less anxiety and depression.

As the social cure tradition shows that identifying with multiple groups (typically those not subject to stigma or discrimination) is associated with better well-being for individuals facing life stressors, we predict that identification as a prisoner will threaten well-being, whereas multiple group memberships will buffer the negative effects group-based rejection on well-being in former inmates. These topics should be explored more accurately within this specific context, considering the perspectives of inmates who are currently in prison.

Specifically, we suggest that the internalization of social stigma is associated with lower perceived well-being. Prisoners who have been discriminated against or who anticipate experiencing discrimination once released will show higher levels of depression, anxiety, and stress. Therefore, we expect to identify a relationship between group-based rejection and well-being.

## 2 Method

### 2.1 Procedure

This study was approved by the Ethical Review Board at the University of Turin (Prot. n. 0565115 del 11/10/2023). Information and consent forms were included at the beginning of the paper-and-pencil questionnaire used in this study.

The present study was carried out at the Rodolfo Morandi Prison, located in the northwest of Italy. This is among the centers mentioned in the report to the Italian Government that does not appear overcrowded and inmates therefore have more suitable space to live and devote themselves to the proposed training and work activities. This is an exclusively male detention center. Data updated (2023), indicate the presence of 354 prisoners, of which only 14 are migrants (rate of crowding: 76.9%; foreign inmates: 3.9%). The building is primarily used to imprison those who are serving sentences for violations of the Criminal Code, related to a Mafia-crimes or to the illicit trafficking of narcotic or psychotropic substances. Currently, a state of overcrowding has not been reported for this facility, and as reported by the outcome of the inspection of the Ministry of Justice (2023), the available spaces, have all proven to be suitable and in good condition.

Regarding educational, cultural, sports, and recreational activities, this prison offers various opportunities to prisoners, who often come from difficult social and family backgrounds. Among the prisoners, 33.3% are reported to be enrolled in school courses.

All participants were men serving their sentences in a High-Security Institute. The type of crime committed was not explored individually as it was insignificant for the purposes of this study.

### 2.2 Sample

52 male participants (14.7% of prisoners) voluntarily replied to the announcement of this study posted on the notice board by educators. They completed a questionnaire with measures of well-being, identification and group membership. 38.4% had middle school diplomas, 32.7% had high school diplomas, and 7.6% had bachelor’s or master’s degrees. Most participants stated that they had continued their studies since entering prison. Furthermore, 21.1% were single, 44.2% were married, 11.5% separated or divorced, and 1.9% were widowers.

### 2.3 Procedure

We used an identity salience manipulation in which the order participants completed the measures differed, with this assigned at random. Some participants completed the measure on identification with the prisoner group first, whereas the others completed the well-being scales before the group identification measure.

Well-being was assessed with measures as Kyprianides et al. (2019), which included the Depression Anxiety Stress Scales—21 items (DASS-21), which is a well-validated short form of the original DASS (Lovibond and Lovibond, 1995). This measure includes three seven-item subscales, assessing depression (*α* = 0.729), anxiety (α = 0.827), and stress symptoms (α = 0.729), respectively. The DASS-21 has excellent validity in both clinical and non-clinical samples and a reliability of at least α = 0.88 (Crawford et al., 2009; Henry and Crawford, 2005). Self-regulation was measured using the short form of the Self-Regulation Questionnaire (SRQ, Brown et al., 1999), which consists of 31 items rated on a five-point scale ranging from 1 = “strongly disagree” to 5 = “strongly agree” (α = 0.72).

Following Cruwys and Gunaseelan (2016), we used three items to measure social identification, including “I feel a bond with other people who are incarcerated,” and “Being a person who is incarcerated gives me a good feeling.” Responses were provided on a five-point scale ranging from 1 = “strongly disagree” to 5 = “strongly agree” (Postmes et al., 2013).

Perceived discrimination was measured using an adapted version of the Self-Stigma of Individuals with Criminal Records Scale (SSICR; Moore et al., 2013). The scale assesses perceptions of the attitudes underlying discriminatory behavior, followed by eight statements such as “cannot be trusted” or “are dangerous.” The participants selected those they felt were the most important. Finally, to measure future expectations, the following statement was used: “I think I regularly encounter discrimination against people who have been previously incarcerated” (1 = “not at all” to 6 = “strongly agree”).

## 3 Results

We used G*Power to determine the necessary sample size before data collection (Faul et al., 2009). The power analysis revealed that a minimum sample of *N* = 51 was required to detect a *rho* = 0.3, assuming an *α* = 0.05 and power = 0.70 for mean comparisons between two groups (first and last identification conditions). We used bootstrapping to evaluate statistical significance through 1,000 repeated samples to construct Monte Carlo 95% confidence intervals and assess the significance of the tau-B correlation between social identification and DASS scores.

We used Student’s *t*-test to compare perceptions of stigma toward prisoners and self-stigma. The mean score showed the salience of identification and more slightly with life satisfaction among the respondents: when participants completed the identification items first, they tended to show higher identification with the prisoner group (M = 3.679 vs. M = 3,244 *t* = 2.125, *p* < 0.005). The effect size, as measured by Cohen’s *d*, was *d* = 0.589, indicating a medium effect.

The results of a χ 2 analysis showed a correspondence in attribution between self and hetero-assessment attesting to the relevance of perceived discrimination (χ 2 = 0.822, *p* < 0.001). The characteristics that respondents identified as being most frequently attributed to prisoners and, as such, to themselves, confirm the dehumanization typical of stigma (Table 1). The results of an ANOVA showed differences in scores for identification with the group and self-regulation between those who had received an educational qualification in prison (bachelor’s or master’s degree or diploma) and those who had a secondary school diploma. In particular, regarding the ability to self-regulate, those who had a university degree (M = 3.92) or diploma (M = 3.59) scored higher [*F*(2, 40) = 4.326, *p* < 0.05, ŋ^2^ = 0.19] than those with a secondary school certificate (M = 3.37). Furthermore, regarding identification with the prisoner group, those who had a diploma (M = 3.76) showed more identification than those who had a degree (M = 2.75), *t* = 2,416, *p* < 0.05, *d* = 0,654. Identification with the prisoner group was positively correlated (Kendall’s т) with the DASS subscales of stress (*r* = 0.232, 95% BCa CI [0.00, 0.46], *n* = 52, *p* < 0.05), depression (*r* = 0.26, 95% BCa CI [0.04, 0.47], *n* = 52, *p* < 0.05), and anxiety (*r* = 0.29, 95% BCa CI [0.09, 0.48], *n* = 52, *p* < 0.005).

**Table 1:** Descriptive statistics related to other vs. self-perception of stigma.

	The Public believes most people with a criminal record		Because I have a criminal record	
	Others (*N*)	%	Self (*N*)	%
Cannot be trusted	25	49	30	81.1
Are disgusting	10	19.6	1	2.7
Are unwilling to get or keep a regular job	6	11.8	3	8.1
Are dirty and unkempt	2	3.9	2	5.4
Are below average in intelligence	1	2.0	-	-
Are unpredicatable	4	7.8	1	2.7
Cannot be rehabilitated	2	3.9	-	-
Are dangerous	1	2	-	-
Are bad people	25	49	-	-
	51	100	37	100

## 4 Discussion

The findings provide evidence supporting previous research (Kyprianides et al., 2019) showing that perceived discrimination negatively affects well-being. We found that prisoners who identified with stigmatized groups reported more depressive symptoms than those who did not. Simultaneously, the findings on self-regulation process showed that education can have practical implications for changing and better support even where desistance take place. Indeed, the results showed the role that social groups, in a vulnerable population, can play in building a positive identity that helps manage a full and satisfying social life. This has potential significance for policy and practice, should research showed the role of social ties and social interactions in prison.

This study has some limitations. As this study was conducted in Italy, the findings may not be generalizable to other populations. The survey used psychometrically robust questionnaires that can enable future comparisons with our findings on of social group identification among prisoners with others that differ in context and levels of well-being, although a potential response bias is also acknowledged. Further experimental designs are recommended to analyze the psychological process of both identity changing and the influence of education. Given the small sample size and missing data related to respondent characteristics, the results should be viewed with caution. To better understand prisoners’ responses, contextual information should be provided on the type of structure and possibilities offered to prisoners during the rehabilitation process, as these can play a decisive role in the degree of well-being.

The findings indicate that the degree of perceived discrimination also has a strong impact on their well-being. Furthermore, education plays a fundamental role in prisoners’ well-being. Thus, access to comprehensive educational programs is essential for enabling prisoners to experiment with their self-evaluation and explore new identities for promoting their sense of well-being and self-regulation.

## Data Availability

The raw data supporting the conclusions of this article will be made available by the authors, without undue reservation.
